# Alteration of GABAergic Input Precedes Neurodegeneration of Cerebellar Purkinje Cells of NPC1-Deficient Mice

**DOI:** 10.3390/ijms20246288

**Published:** 2019-12-13

**Authors:** Michael Rabenstein, Nico Murr, Andreas Hermann, Arndt Rolfs, Moritz J. Frech

**Affiliations:** 1 Translational Neurodegeneration Section “Albrecht-Kossel”, Department of Neurology, University Medical Center Rostock, University of Rostock, 18147 Rostock, Germany; m.rabenstein@uni-bonn.de (M.R.); nico.murr@uni-rostock.de (N.M.); andreas.hermann@med.uni-rostock.de (A.H.); 2 Center for Transdisciplinary Neurosciences Rostock (CTNR), Rostock University Medical Center, University of Rostock, 18147 Rostock, Germany; 3 CENTOGENE AG, 18055 Rostock, Germany; arndt.rolfs@centogene.com

**Keywords:** NPC1, inhibitory synaptic transmission, patch clamp, AMPA-receptor

## Abstract

Niemann-Pick Disease Type C1 (NPC1) is a rare hereditary neurodegenerative disease belonging to the family of lysosomal storage disorders. NPC1-patients suffer from, amongst other symptoms, ataxia, based on the dysfunction and loss of cerebellar Purkinje cells. Alterations in synaptic transmission are believed to contribute to a pathological mechanism leading to the progressive loss of Purkinje cells observed in NPC1-deficient mice. With regard to inhibitory synaptic transmission, alterations of GABAergic synapses are described but functional data are missing. For this reason, we have examined here the inhibitory GABAergic synaptic transmission of Purkinje cells of NPC1-deficient mice (NPC1^−/−^). Patch clamp recordings of inhibitory post-synaptic currents (IPSCs) of Purkinje cells revealed an increased frequency of GABAergic IPSCs in NPC1^−/−^ mice. In addition, Purkinje cells of NPC1^−/−^ mice were less amenable for modulation of synaptic transmission via the activation of excitatory NMDA-receptors (NMDA-Rs). Western blot testing disclosed a reduced protein level of phosphorylated alpha-amino-3-hydroxy-5-methyl-4-isoxazolepropionic acid receptors (AMPA-Rs) subunit GluA2 in the cerebella of NPC1^−/−^ mice, indicating a disturbance in the internalization of GluA2-containing AMPA-Rs. Since this is triggered by the activation of NMDA-Rs, we conclude that a disturbance in the synaptic turnover of AMPA-Rs underlies the defective inhibitory GABAergic synaptic transmission. While these alterations precede obvious signs of neurodegeneration of Purkinje cells, we propose a contribution of synaptic malfunction to the initiation of the loss of Purkinje cells in NPC1. Thus, a prevention of the disturbance of synaptic transmission in early stages of the disease might display a target with which to avert progressive neurodegeneration in NPC1.

## 1. Introduction

Niemann Pick Type C1 (NPC1) is a recessive inherited neurovisceral lipidosis with an estimated incidence of 1:120,000 [1]. Mutations in the NPC1 gene lead to an accumulation of mainly cholesterol, gangliosides, and sphingosines in lysosomes/late endosomes [2]. Patients present, amongst other symptoms, neurological symptoms such as cerebellar ataxia, which is caused by a progredient loss of Purkinje cells (PCs) [2]. Studies in NPC1 mice models have revealed progressive cell loss propagating from the anterior to the posterior lobes, whereas no prominent loss of PCs has been observed in lobe 10 [3,4,5]. The onset of loss of PCs in (the here-used) NPC1^−/−^ mice becomes obvious at 4–5 weeks of age and severe motor deficits can be recognized at 6–7 weeks of age [3,4,6,7]. The pathological mechanism underlying the loss of PCs is not yet well understood and only a few studies describe functional alterations in PCs of NPC1^−/−^ mice at an age without any obvious signs of cell loss or motoric dysfunction. Cerebellar PCs represent the sole afferent neurons in the cerebellar cortex and act as a pacemaker by generating intrinsic action potentials (APs). The AP firing patterns underlie a modulation of excitatory and inhibitory synapses. An increased frequency of excitatory post-synaptic currents has been, e.g., described in mice with an age ranging from p17 to p23 [8]. With regard to inhibitory synaptic transmission mediated by gamma-aminobutyric acid receptor (GABA_A_-Rs), an altered number of GABAergic synapses has been described in NPC1-deficient mice, but functional data are not available [9]. Thus, we examined inhibitory postsynaptic currents (IPSCs), mediated by GABA_A_-Rs, to elucidate alterations of the synaptic transmission between cerebellar interneurons and Purkinje cells. Since we have recently reported a disturbed internalization of alpha-amino-3-hydroxy-5-methyl-4-isoxazolepropionic acid receptors (AMPA-Rs) containing the GluA2 subunit [10,11] and that the GABAergic input to the PCs can be tuned by N-methyl-D-aspartate receptors (NMDA-Rs) and subsequently by AMPA-Rs [12], we examined in addition the modulation of the synaptic input to PCs by activating NMDA-Rs and the expression of the GluA2 subunit of AMPA-Rs.

## 2. Results

### 2.1. GABAergic Input to Purkinje Cells Is Altered in NPC1^−/−^ Mice

Progressive loss of Purkinje cells is a hallmark of NPC1, wherein different NPC1 mice models show progressive cell loss propagating from the anterior to posterior lobes [3,4,5]. In (the here-used) NPC1^−/−^ mice, the loss of PCs can be observed at 4–5 weeks of age, followed by motor deficits at 6–7 weeks of age [3,4,6,7]. Only a few studies have described functional alterations in PCs of NPC1^−/−^ mice at an age without any obvious signs of cell loss or motoric dysfunction. An elevated frequency of excitatory post synaptic currents has been, e.g., described in mice with an age ranging from p17 to p23 [8]. Since no data concerning inhibitory synaptic transmission to PCs in NPC1^−/−^ mice at this age are available, we studied GABAergic transmission in NPC1^−/−^ and NPC1^+/+^ mice in the age range p19 to p25.

**Figure 1:**
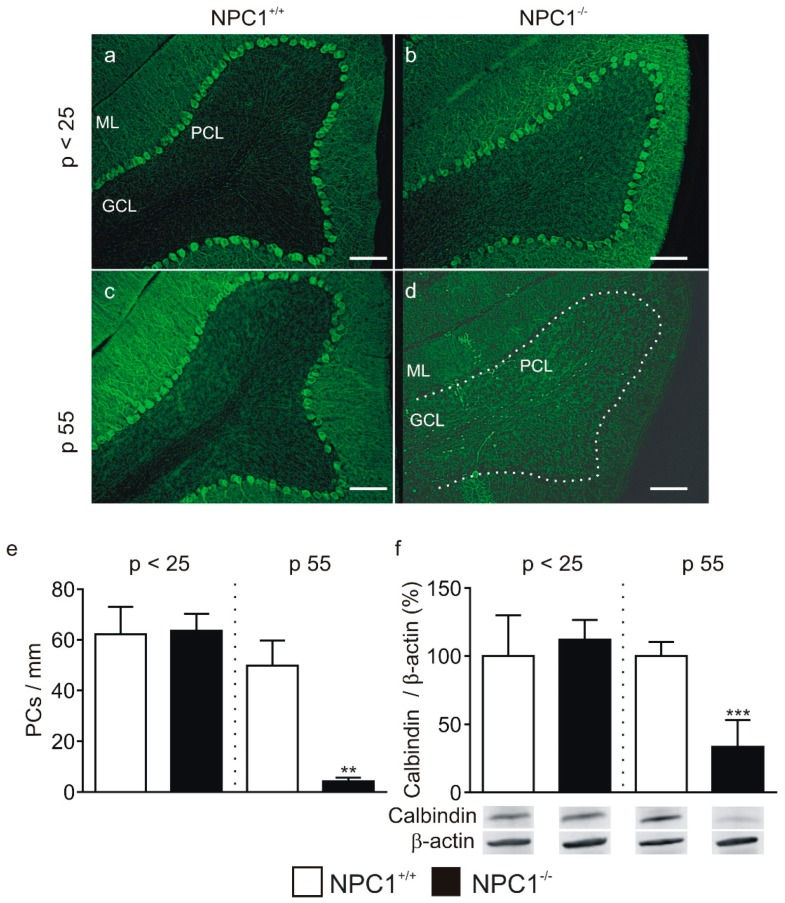
Purkinje cell (PC) degeneration in Niemann-Pick Disease Type C1 (NPC1)^−/−^ mice. (**a**–**d**) Loss of Purkinje cells in lobe III of NPC1^−/−^ mice was observed at p 55, but not at *p* < 25 in stainings against calbindin D28K. (**e**) Significantly less Purkinje cells per mm were present in lobe III of p 55 NPC1^−/−^ mice. (**f**) Western blot analysis of cerebellar lysates showed a significant reduction in cerebellar calbindin levels in p 55 but not in *p* < 25 NPC1^−/−^ mice. Western blot bands display corresponding examples of the same gel of Western blot. The protein level of NPC1^+/+^ mice was set as 100%. ** *p* < 0.01, *** *p* < 0.001. Legend: ML, molecular layer; PCL, Purkinje cell layer; GCL, granular cell layer. The dashed line in (d) marks the PCL. Scale bar indicates 100 µm.

Immunofluorescence stainings, using calbindin as a marker for PCs, showed no difference in the PC density between *p* < 25 NPC1^+/+^ and NPC1^−/−^ mice. Both genotypes displayed an intact PC layer, e.g., in lobe III (Figure 1a,b) and no differences in PC density (NPC1^+/+^: 62 ± 8 PCs/mm, NPC1^−/−^: 64 ± 5 PCs/mm, N = 2–3, *n* = 2, Figure 1e). Comparable results were obtained by Western blot analysis of calbindin using whole cerebellar lysates, showing no differences in the protein level between *p* < 25 NPC1^+/+^ and NPC1^−/−^ mice (NPC1^+/+^: 100 ± 12%, NPC1^−/−^: 112 ± 6%, N = 2–3, *n* = 6, *p* = 0.391, Figure 1f). The same analysis performed with p 55 animals affirmed a prominent loss of PCs (Figure 1c,d). NPC1^−/−^ mice displayed a disrupted PC layer and a significant loss of PCs (NPC1^+/+^: 49 ± 6 PCs/mm, NPC1^−/−^: 4 ± 1 PCs/mm, N = 2–3, *n* = 3, *p* = 0.001, Figure 1e), accompanied by a significantly reduced calbindin protein level (NPC1^+/+^: 100 ± 5%, NPC1^−/−^: 33 ± 10%, N = 2–3, *n* = 4, *p* ≤ 0.001, Figure 1d–f).

**Figure 2:**
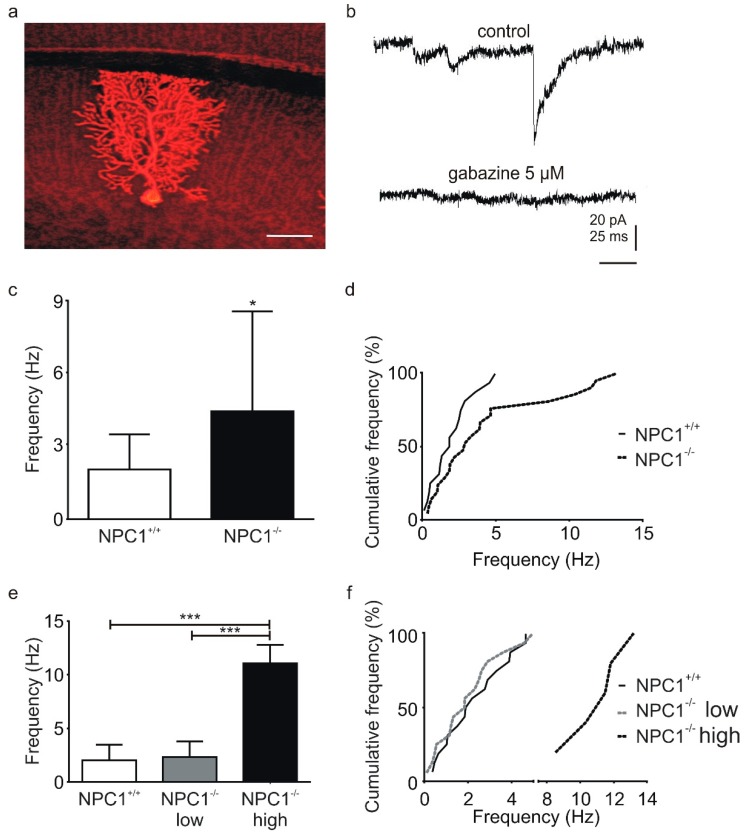
Increased basal inhibitory post-synaptic current (IPSC) frequency in NPC1^−/−^ mice. (**a**) PCs were filled with Neurobiotin™ during the patch clamp recordings of IPSCs and subsequently visualized by Texas Red^®^ streptavidin. The example represents a PC recorded in a parasagittal cerebellar slice of an NPC1^+/+^ mouse. (**b**) Example of a recording of postsynaptic currents in PCs (upper trace). Application of gabazine-abolished IPSCs confirmed that the IPSCs were mediated by gamma-aminobutyric acid receptor (GABA_A_-Rs). (**c**,**d**) Analysis of IPSC frequencies. The frequency of IPSCs was significantly increased in Purkinje cells of NPC1^−/−^ mice (c), wherein the cumulative plot of the relative frequency revealed a second population of IPSCs occurring with a higher frequency (d). (**e**,**f**) Analysis of subpopulations of IPSCs. The division of the IPSCs of NPC1^−/−^ mice showed no significant difference between NPC1^+/+^ mice and the NPC1^−/−^ low frequency group, but a significantly higher IPSC frequency in the NPC1^−/−^ high frequency group. * *p* < 0.05, *** *p* < 0.001. Scale bar indicates 25 µm.

To study the GABAergic transmission, we used mice between p19 and p25 which displayed an intact PC layer without any obvious signs of loss of PCs or degeneration of the cells. Parasagittal cerebellar slices, containing PCs with an intact dendritic tree, were used to record IPSCs (Figure 2a). IPSCs were completely abolished by the GABA_A_-R antagonist gabazine, confirming that the IPSCs were mediated by GABA_A_-R (Figure 2b).

A comparison of the control phases revealed that the basal IPSC frequency was significantly higher in NPC1^−/−^ mice (NPC1^+/+^: 2.0 ± 1.4 Hz, NPC1^−/−^: 4.4 ± 4.1 Hz, N = 7–11, *n* = 16–21, *p* = 0.041, Figure 2c (left)). The high standard deviation of these data sets and the cumulative frequency distribution indicated an additional group of Purkinje cells with an IPSC frequency higher than 5 Hz in NPC1^−/−^ mice (Figure 2c (right)). Thus, we separated the PCs using a cut-off frequency of four times the standard deviation of the mean IPSC frequency of the NPC1^+/+^ mice (5.8 Hz). Consequently, PCs of the NPC1^−/−^ mice were grouped with PCs with low IPSC frequencies (NPC1^−/−^ low) and high frequencies (NPC1^−/−^ high, Figure 2e). A comparison between the mean frequency and distribution of the NPC1^−/−^ low-frequency group with mean frequency of the NPC1^+/+^ mice showed no significant difference (NPC1^−/−^: 2.3 ± 1.4 Hz, NPC1^+/+^: 2.0 ± 1.4 Hz, N = 10, *n* = 16, *p* = 0.923, Figure 2e). However, the frequency of the NPC1^−/−^ high frequency group (NPC1^−/−^ high: 11.1 ± 1.7 Hz, N = 5, *n* = 5) was significantly higher than that of the NPC1^+/+^ or the NPC1^−/−^ low frequency group (Figure 2e).

**Figure 3:**
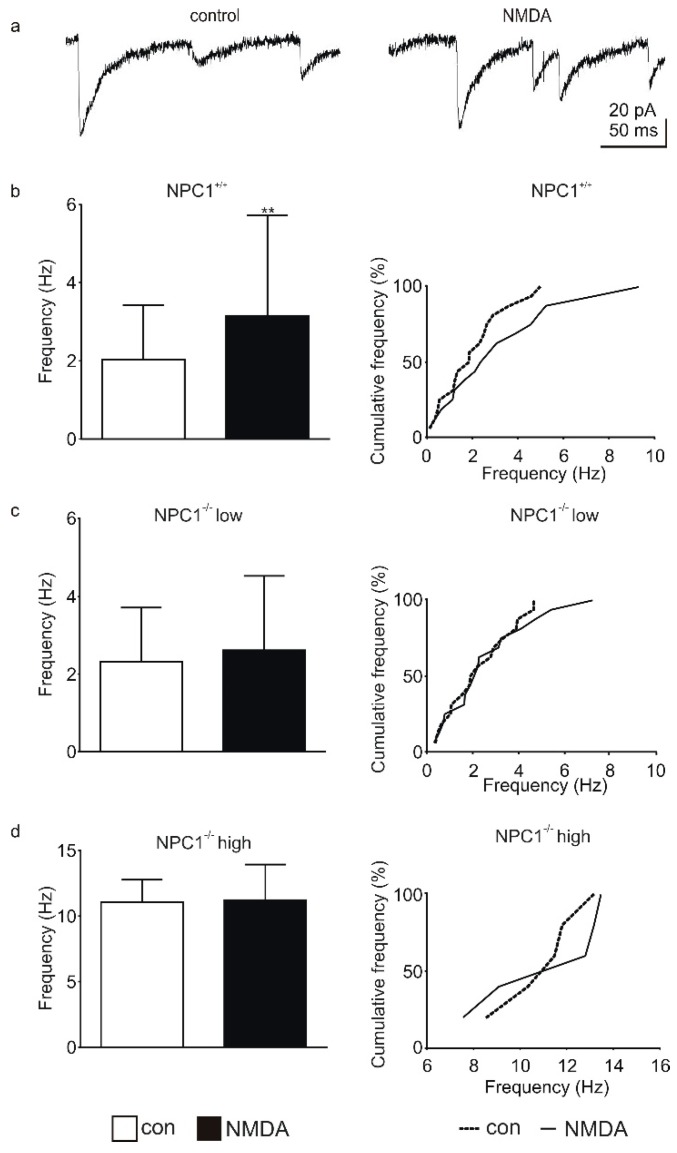
Altered GABAergic synaptic transmission and modulation to Purkinje cells in NPC1^−/−^ mice. (**a**) Example of recording of IPSCs under control conditions (control) and the presence of NMDA (NMDA). (**b**) The frequency of IPSCs increased significantly during NMDA application in NPC1^+/+^ mice. (**c**,**d**) No significant increase in IPSC frequency was observed in the NPC1^−/−^ low group as well as in the NPC1^−/−^ high group during NMDA application. ** *p* < 0.01.

Purkinje cells receive GABAergic synaptic inputs from basket and stellate cells [12], for which this inhibitory input can be modulated by NMDA-Rs, which are located on the interneurons themselves [12,13]. Since an application of NMDA can trigger an increased frequency of GABAergic IPSCs (Figure 3a) [12,13], we recorded IPSCs in the presence of NMDA. Examples of a control recording of IPSCs and IPSCs recorded in the presence of NMDA are shown in Figure 3. In NPC1^+/+^ mice the application of NMDA significantly increased the IPSC frequency (control: 2.0 ± 1.4 Hz, NMDA: 3.2 ± 2.6 Hz, N = 7, *n* = 16, *p* = 0.007, Figure 3b). By contrast, in NPC1^−/−^ mice no increase was observed in the low-frequency group (control: 2.3 ± 1.4 Hz, NMDA: 2.6 ± 1.9 Hz, N = 10, *n* = 16, *p* = 0.633, Figure 3c) as well as in the high-frequency group (control: 11.1 ± 1.7 Hz, NMDA: 11.2 ± 2.7, N = 5, *n* = 5, *p* = 0.691, Figure 3d).

In summary, the basal IPSC frequency was increased in a subpopulation of Purkinje cells of NPC1^−/−^ mice and NMDA failed to raise the basal IPSC frequency in NPC1^−/−^ mice. The NMDA-induced modulation of the IPSC frequency is most likely based on an increased expression of GluA2-containing AMPA-Rs located in the synapses of the parallel fiber interneurons [12]. The activation of NMDA-Rs is believed to trigger the exchange of AMPA-Rs, inducing a potentiation of the glutamatergic synapses and resulting in an increased release of GABA [12]. The expression and phosphorylation of the AMPA-R subunit GluA2 is believed to play a crucial role in synaptic plasticity [14]. Recently, we described an altered expression and phosphorylation of GluA2 in cortical neurons of NPC1^−/−^ mice [10] and in NPC1-deficient neurons derived from NPC1 patient-specific induced pluripotent stem cells [11]. Hence, we checked in a next step the expression and phosphorylation of GluA2 in the cerebella of NPC1^−/−^ mice.

### 2.2. The AMPA-Receptor Subunit GluA2 Is Hypophosphorylated in the Cerebella of NPC1^−/−^ Mice

The AMPA-R subunit exchange plays a significant role in the modulation of synaptic transmission, which in turn depends on phosphorylation. The phosphorylation of the Ser880 site of GluA2 is, e.g., mediated by protein kinase C (PKC) [15,16]. Moreover, a hampered function of PKC has been described as occurring in NPC1-deficient cells [15,16,17,18,19]. Consequently, the different effects of NMDA in NPC1^+/+^ and NPC1^−/−^ mice could be based on an altered expression or phosphorylation of AMPA-Rs. Hence, we analyzed the protein levels of GluA2 and GluA2 phosphorylated at the Ser880 site (p-GluA2), representing the internalized form of GluA2 [15,16].

**Figure 4:**
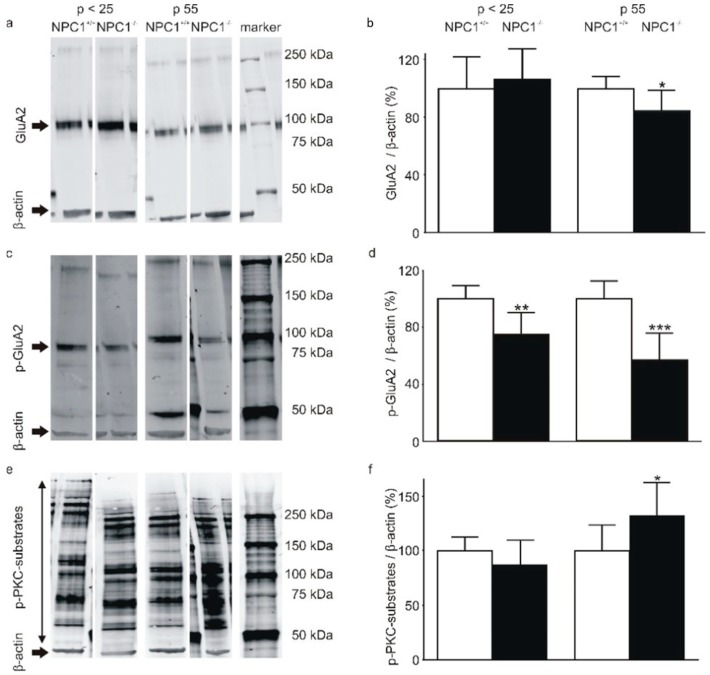
Altered protein kinase C (PKC)-dependent phosphorylation of cerebellar proteins in NPC1^−/−^ mice. (**a,b**) A significantly reduced cerebellar GluA2 level was observed in p 55 NPC1^−/−^ mice but not in *p* < 25 mice. (**c,d**) The protein level of Ser880-phosphorylated GluA2 (p-GluA2) was significantly reduced in *p* < 25 and p 55 NPC1^−/−^ mice. (**e,f**) No significant change in the protein level of phosphorylated PKC-substrates (PKC-substrate) was observed in *p* < 25 NPC1^−/−^ mice. In p 55 mice the protein level of PKC-substrates was significantly increased. The protein level of NPC1^+/+^ mice was set as 100%. * *p* < 0.05, ** *p* < 0.01, *** *p* < 0.001.

In cerebellar lysates of *p* < 25 mice, the total amount of GluA2 was not significantly different between NPC1^+/+^ and NPC1^−/−^ mice (NPC1^+/+^: 100 ± 23%, NPC1^−/−^: 107 ± 21%, N = 3, *n* = 6, *p* = 0.463, Figure 4a,b). By contrast, in p 55 mice the total amount of GluA2 in NPC1^−/−^ mice was significantly lower compared to NPC1^+/+^ mice (NPC1^+/+^: 100 ± 8%, NPC1^−/−^: 85 ± 13%, N = 3, *n* = 6, *p* = 0.002, Figure 4a,b). The protein level of p-GluA2 was significantly lower in *p* < 25 NPC1^−/−^ mice (NPC1^+/+^: 100 ± 9%, NPC1^−/−^: 75 ± 15%, N = 3, *n* = 6, *p* < 0.001) and p 55 NPC1^−/−^ mice (NPC1^+/+^: 100 ± 12%, NPC1^−/−^: 57 ± 19%, N = 3, *n* = 6, *p* < 0.001, Figure 4c,d).

To assess the contribution of reduced PKC activity, an antibody against phosphorylated serines in the PKC target sequence (R/K)X(S*)(Hyd)(R/K) was utilized for Western blot analysis of phosphorylated PKC substrates. Since the antibody detected phosphorylated serines in a variety of proteins, bands located upper to the β-actin band were quantified as p-PKC-substrates (Figure 4e). The signal of the p-PKC-substrates was not significantly different between *p* < 25 NPC1^+/+^ and NPC1^−/−^ mice (NPC1^+/+^: 100 ± 13%, NPC1^−/−^: 89 ± 23%, N = 3, *n* = 6, *p* = 0.172, Figure 4f). By contrast, in p 55 NPC1^−/−^ mice the amount of p-PKC-substrates was significantly elevated (NPC1^+/+^: 100 ± 23%, NPC1^−/−^: 132 ± 30 %, N = 3, *n* = 6, *p* = 0.008), indicating an increase in PKC activity (Figure 4f).

Taken together, *p* < 25 NPC1^+/+^ and NPC1^−/−^ mice demonstrated a comparable amount of total GluA2 but reduced p-GluA2 protein levels. This hints at an increased amount of membrane bound GluA2, which is also present in p 55 NPC1^−/−^ mice. Contrary to the conclusions of former publications, a reduced PKC activity was not determined when using an antibody against p-PKC-substrates. By contrast, in p 55 NPC1^−/−^ mice, an increased amount of p-PKC-substrates was measured, indicating a PKC hyperactivity, possibly as a compensatory mechanism.

## 3. Discussion

Ataxia, a pathological hallmark of NPC1, is caused by dysfunction and progressive loss of Purkinje cells, displaying the sole output of the cerebellar cortex [2]. In this context, the aim of this study was to investigate whether functional alterations can be detected in GABAergic synaptic transmission to Purkinje cells in NPC1^−/−^ mice in advance of degeneration of PCs. Using voltage clamp recordings, we identified an increased IPSC frequency in a subset of Purkinje cells in NPC1^−/−^ mice, indicating a higher activity of the presynaptic interneurons. Independent of the increased IPSC frequency, we revealed an impaired modulation of NMDA-induced alteration of GABAergic synaptic transmission in NPC1^−/−^ mice. To determine the underlying molecular mechanism, we compared the cerebellar protein levels of the AMPA-R subunit GluA2 and its Ser880-phosphorylated form. Since the phosphorylation of GluA2 at Ser880 leads to the internalization of AMPA-R [15,16], the reduced relative amount of p-GluA2 we found in NPC1^−/−^ mice hints to an increased surface expression of GluA2-positive AMPA-R. This is in line with results regarding the cortical neurons of NPC1^−/−^ mice [10] and human NPC1-deficient neurons, derived from human-induced pluripotent stem-cells, carrying mutations in the NPC1 gene [11]. NMDA has been observed to induce an increase in synaptically-located GluA2-containing AMPA-Rs in cerebellar interneurons [12]. Thus, we conclude that the inability of NMDA to induce an increase in IPSC frequency in NPC1^−/−^ mice is based on hampered exchange of AMPA-Rs. Subsequently, the activity of interneurons is likely to be increased, resulting in an elevated GABAergic synaptic transmission to the postsynaptic Purkinje cells, and consequently not able to be further elevated by NMDA. In addition, a dysfunction of KCNQ1/2 leading to hyperexcitability of NPC1-deficient neurons might contribute to this effect [20].

Our findings are in accordance with studies reporting alterations in excitatory synaptic transmission in NPC1. An increased frequency of miniature excitatory postsynaptic currents has been reported in hippocampal CA1 pyramidal cells and Purkinje cells [8,21]. Additionally, the plasticity of excitatory synaptic transmission has been described as impaired. In neocortical neurons impaired long-term potentiation and in Purkinje cells impaired long-term depression have been found in NPC1-deficient mice [8,21,22]. In the hippocampal CA1 region AMPA has been observed to fail to decrease field potentials [23]. Based on these studies it is likely that the excitatory synaptic transmission is trapped at a high level in NPC1 due to an impaired AMPA-R exchange.

A reason for this might be given by an altered PKC activity, since PKC-dependent phosphorylation is important for AMPA-R subunit exchange, e.g., the internalization of GluA2-containing AMPA-Rs upon phosphorylation of the Ser880 site [15,16]. In previous studies PKC hypoactivity has been proposed to be present in NPC1-mutated cells [17,18,19]. This assumption is based on a reduced amount of the phosphorylated forms of vimentin and the glial fibrillary acidic protein (GFAP), which are PKC substrates. Furthermore, pharmacological activation of PKC has been observed to correct the altered vimentin and GFAP phosphorylation and reduce the intracellular cholesterol accumulations in NPC1-deficient cells [17,18,19]. Interestingly, when using an antibody against phosphorylated PKC substrates in general, we did not find a reduced amount of phosphorylated proteins in NPC1^−/−^ mice. By contrast, we observed an increased amount of phosphorylated PKC substrates in p 55 NPC1^−/−^ mice.

This conflictive observation might be explained by differences in PKC dysfunctions in diverse cell compartments. A cell-compartment-specific dysfunction of PKC has, for example, been described for spinocerebellar ataxia type 14 (SCA14), which is caused by mutations in the PKCγ gene [24]. Certain mutations of the PKCγ gene impact the retention time of PKCγ in the membrane, resulting in a decreased activity of membrane-bound PKCγ and an increased activity of PKCγ in the cytosol [24]. With regard to NPC1, a disturbed cholesterol homeostasis could alter the lipid composition of, e.g., lipid rafts in the cell membrane, as has been recently reported [10], thus impacting the retention time of PKC. However, further experiments are warranted to prove an impact of translocation problems of PKC to the pathogenic mechanisms of NPC1.

## 4. Materials and Methods

### 4.1. Animal Housing

Heterozygous BALB/c_Nctr-Npc1m1N/-J mice (Jackson Laboratories, Bar Harbor, ME, USA) [4] were mated to obtain homozygous NPC1-deficient (NPC1^−/−^) and unaffected wild-type (NPC1^+/+^) animals. Mice were kept in a 12 h light/dark cycle with access to food and water ad libitum. The genotype of the animals was determined by polymerase chain reaction using tail tip samples. Animals were sacrificed between postnatal day 19 (p19) and p25 and at p 55. The two groups were labelled *p* < 25 and p 55, respectively. Housing and breeding of animals, and experimental procedures were done in accordance with the German Animal Welfare Law (Deutsches Tierschutzgesetz). Approval was given by Landesamt für Landwirtschaft, Lebensmittelsicherheit und Fischerei Mecklenburg-Vorpommern (LALLFMV), Rostock, Germany (19 January 2016).

### 4.2. Preparation of Cerebellar Slices

Preparation of parasagittal cerebellar vermis slices was performed as described recently [25]. Mice were decapitated and brains were removed rapidly and transferred to an ice-cold buffer (buffer 1) consisting of (mM): NaCl 125, KCl 2.5, CaCl_2_H_2_O 2, MgCl_2_∙6H_2_O 1, NaHCO_3_ 26, NaH_2_PO_4_∙H_2_O 1.25, and glucose∙H_2_O 25, with pH being adjusted to 7.4. Two-hundred-and-fifty-micrometer-thick slices were cut with a vibratome (Leica VT 1200S) and subsequently incubated in buffer 1 at 37 °C. All steps were performed with buffer supplied with carbogen (95% O_2_, 5% CO_2_).

### 4.3. Patch Clamp Recordings

Patch clamp recordings were undertaken with an EPC-10 amplifier (Heka, Germany) using the Patchmaster software package (Heka, Lambrecht, Martinsried, Germany). A DMZ-Universal-Electrode-Puller (Zeitz, Germany) was used to pull borosilicate glass pipettes (GC150F-10, Harvard Apparatus, Holliston, MA, USA). The extracellular solution used for patch clamp recordings contained (mM): NaCl 151, KCl 2.5, HEPES 10, CaCl_2_∙H_2_O 2, MgCl_2_∙6H_2_O 1, NaH_2_PO_4_∙H_2_O 1.25, and glucose∙H_2_O 25. The pH was adjusted to 7.4 with NaOH. The intracellular solution contained (mM): CsCl 125, TEA-Cl 20, HEPES 10, EGTA 0.5, MgCl_2_∙6H_2_O 2, Na_2_-ATP 2, and Na-GTP 0.5, 0.1% Neurobiotin™ (Vector Laboratories, Burlingame, CA, USA). The pH was adjusted to 7.3 with CsOH. The electrodes had a resistance of 2–4 MΩ. Recordings of IPSCs of optically-identified Purkinje cells in cerebellar lobes III-V were made in the whole cell configuration in voltage clamp mode with a holding potential of −70 mV at room temperature. To validate the impact of NMDA-receptor activation on synaptic activity, 20 µM NMDA was applied in a subset of experiments using the Octaflow™-System (ALA Scientific Instruments, Farmingdale, NY, USA). Gabazine (5 µM) was used to abolish GABAergic IPSCs in a subset of experiments. Data were filtered at 3 kHz and digitized with 10 kHz. MiniAnalysis 6.0.7 (SynaptoSoft, Fort Lee, NJ, USA) was used to detect post-synaptic currents. Mean basal frequency was calculated from a five-minute time period using R 3.5.1 (The R Foundation for Statistical Computing, Vienna, Austria) with RStudio 1.1.456 (RStudio, Inc., Boston, MA, USA).

### 4.4. Sample Preparation for Western Blot Analysis

Frozen cerebella were used for protein extraction. Tissue was ground in 1 mL RIPA-lysis buffer containing in mM: TRIS 20, NaCl 137, sodium deoxycholate 12, EDTA 2, 0.1% SDS, 1% Triton^®^ X-100, and 10% glycerol supplemented with cOmplete™, mini, EDTA-free protease inhibitor cocktail (Roche Diagnostics GmbH, Mannheim, Germany). Subsequently, samples were incubated on ice for 30 min and centrifuged (15,000× *g*) for 30 min at 4 °C. Protein concentrations were determined with the Pierce™ BCA protein assay kit (Thermo Fisher Scientific, Waltham, MA, USA) according to the supplier´s manual. Next, samples were boiled for 10 min at 95 °C in 5 × Laemmli-buffer (125 mM TRIS, 20% glycerol, 2% SDS, 5% β-mercaptoethanol, 10% bromphenol blue) and centrifuged at 17,530× *g* for 1 min at 4 °C. Criterion™ Vertical Electrophoresis Cell with Criterion™ TGX Stain-Free™ Precast Gels (4–15%) (Bio-Rad Laboratories, Hercules, CA, USA) were used for protein separation. The electrophoresis buffer contained 25 mM TRIS, 200 mM glycine, and 0.1% SDS. Western blot analysis was performed with the Trans-Blot^®^ Turbo™ Transfer System with Trans-Bolt^®^ Turbo™ Transfer Pack (Bio-Rad Laboratories, Germany). Membranes were washed in TRIS-buffered saline (TBS) consisting of 20 mM TRIS and 137 mM NaCl, pH 7.5, for 5 min. Five percent bovine serum albumin (BSA) in TBS supplemented with 0.1% Tween^®^ 20 (TBST) was used for blocking. Membranes were then incubated with primary antibody solution (3% BSA in TBST) for 1 h, washed three times with TBST, and incubated with DyLight™ secondary antibody for 1 h. Membranes were finally washed three times with TBST and once with TBS and dried. The Odyssey Infrared Imaging System (LI-COR Biosciences GmbH, Bad Homburg vor der Hoehe, Germany) was used for semi-quantitative analysis of the protein amount. Antibodies used for Western blot were GluR-2 (N19) (1:200, Santa Cruz Biotechnology, Dallas, TX, USA), Calbindin D28K (1:500, Santa Cruz Biotechnology, Dallas, TX, USA), anti-ionotropic glutamate receptor 2 (phospho-S880) antibody (1:1500, Abcam, Cambridge, UK), Phospho-(Ser) PKC substrate (1:1000, Cell Signaling Technology, Danvers, MA, USA), β-actin (1:10,000, Sigma-Aldrich, St. Louis, MO, USA), anti-rabbit IgG (H&L), DyLight™ 680 (1:10,000), anti-mouse IgG (H&L), and DyLight™ 800 (1:10,000, Rockland Immunochemicals Inc., Limerick, PA, USA). As a molecular weight marker Precision Plus Protein Dual Xtra Standards (Bio-Rad Laboratories, Hercules, CA, USA) were used.

### 4.5. Immunocytochemistry

For calbindin stainings, cryo-thin sections of perfusion-fixed animals were prepared. Briefly, animals were killed with an overdose of sevoflurane. Animals were first perfused with 5 mL ice-cold 0.9% NaCl solution and afterwards fixed with 50 mL 3.7% paraformaldehyde in 0.1 M phosphate-buffered saline (PBS). Perfused brains were isolated and afterwards fixed overnight in 50 mL paraformaldehyde solution at 4 °C. Subsequently, brains were incubated overnight in 50 mL 20% sucrose in PBS at 4 °C, frosted in −80 °C cold 2-methylbutane and, stored at −80 °C.

Parasagittal cerebellar slices (30 µm) were stained free-floating. Sections were washed three times with TBST. Antigen retrieval was performed by incubating the sections for 30 min at 95 °C in a citrate buffer (10 mM tri-sodium citrate dehydrate (pH 6 with HCl) + 0.05% Tween^®^ 20). Sections were blocked with 10% normal goat serum (NGS) in TBST + 0.2% Triton^®^ X-100 for 30 min under agitation at room temperature. Sections were incubated with anti-Calbindin D28K antibody (1:500, Synaptic Systems, Germany) in 1% NGS in TBST for 1 h under agitation at room temperature and afterwards washed with TBST. Then, sections were incubated with secondary antibody (goat anti-chicken IgY (H+L) secondary antibody, Alexa Fluor 488, 1:1000, Thermo Fisher Scientific, Waltham, MA, USA) in 1% NGS in TBST for 1 h under agitation at room temperature, and afterwards washed with TBST and PBS. Sections were mounted in Mowiol-DABCO (10% Mowiol^®^ 4-88, 2.5% DABCO (1,4-diazabicyclo[2.2.2]octane), 25% glycerol, and 0.1 M Tris-HCl (pH 8.5)).

Acute cerebellar brain slices were fixed with 4% paraformaldehyde (PFA) in PBS overnight at 4 °C. Purkinje cells were filled with Neurobiotin™ during patch clamp recordings and subsequently visualized using the protocol provided by Abcam [26]. Fixed slices were washed with TBS-Triton (50 mM TRIS and 150 mM NaCl, pH 7.5, supplemented with 1% Triton^®^ X-100). Subsequently, slices were blocked with 4% NGS in TBS-Triton for 1 h under agitation at room temperature. To visualize Neurobiotin™ in recorded Purkinje cells, slices were incubated in 5 µg/mL Texas Red^®^ streptavidin (Vector Laboratories, Burlingame, CA, USA) in 1% NGS, and TBS-Triton was added for 1 h at room temperature. Finally, slices were washed and mounted in Mowiol-DABCO. The z-stack function of a BZ-8000K microscope (KEYENCE, Germany) was used to obtain pictures of Purkinje cells. The Full Focus function of Analyzer software (KEYENCE, Germany) was used to merge the single pictures.

### 4.6. Statistical Analysis

GraphPad Prism 6.07 (GraphPad Software Inc., San Diego, CA, USA) was used to analyze the data. Data have been given as mean ± SD. Data were tested for normality using the D’Agostino-Pearson normality test. An unpaired Student’s *t*-test and paired Student’s *t*-test or one-way ANOVA with adjusted *p* values were used to determine statistical significance in normally distributed data. The Mann-Whitney test or Dunn’s multiple comparison test were used for not normally distributed data. *p* values < 0.05 were considered statistically significant, with * *p* < 0.05, * *p* < 0.01, and *** *p* < 0.001. N stood for the number of animals; n stood for the number of individual experiments.

## 5. Conclusions

The pathogenic mechanisms underlying the neurodegeneration of Purkinje cells, observed in NPC1, remain elusive. However, accumulating data suggests a contribution of alterations in excitatory synaptic transmission to Purkinje cell loss, preceding obvious signs of neurodegeneration. In accordance with reported changes in excitatory synaptic transmission, we have described here an increased frequency of GABAergic IPSCs, reflecting alterations in the inhibitory synaptic transmission to PCs. Moreover, the modulation of this inhibitory input to PCs, based on NMDA-Rs and AMPA-Rs located in the inhibitory presynaptic interneurons, was found to be jammed in NPC1-deficient mice. These findings strengthen the line of evidence which suggests that changes in neuronal networks account for the initiation of the progressive cell loss observed in NPC1. Thus, prevention of the disturbance of synaptic transmission in early stages of the disease might display a target with which to avert progressive neurodegeneration in NPC1.
